# Myofibroblast-derived exosomes enhance macrophages to myofibroblasts transition and kidney fibrosis

**DOI:** 10.1080/0886022X.2024.2334406

**Published:** 2024-04-04

**Authors:** Wenqiang Yu, Jinfang Song, Shuangquan Chen, Jiayi Nie, Chujun Zhou, Jiamin Huang, Hua Liang

**Affiliations:** a Department of Anesthesiology, Foshan Women and Children Hospital, Foshan, China; b Zhuhai Campus, Zunyi Medical University, Zhuhai, China; c Jiangxi University of Traditional Chinese Medicine, Nanchang, China

**Keywords:** Exosomes, macrophages, myofibroblasts, renal fibrosis

## Abstract

A critical event in the pathogenesis of kidney fibrosis is the transition of macrophages into myofibroblasts (MMT). Exosomes play an important role in crosstalk among cells in the kidney and the development of renal fibrosis. However, the role of myofibroblast-derived exosomes in the process of MMT and renal fibrosis progression remains unknown. Here, we examined the role of myofibroblast-derived exosomes in MMT and kidney fibrogenesis. *In vitro*, transforming growth factor-β1 stimulated the differentiation of kidney fibroblasts into myofibroblasts and promoted exosome release from myofibroblasts. RAW264.7 cells were treated with exosomes derived from myofibroblasts. We found purified exosomes from myofibroblasts trigger the MMT. By contrast, inhibition of exosome production with GW4869 or exosome depletion from the conditioned media abolished the ability of myofibroblasts to induce MMT. Mice treatment with myofibroblast-derived exosomes (Myo-Exo) exhibited severe fibrotic lesion and more abundant MMT cells in kidneys with folic acid (FA) injury, which was negated by TANK-banding kinase-1 inhibitor. Furthermore, suppression of exosome production reduced collagen deposition, extracellular matrix protein accumulation, and MMT in FA nephropathy. Collectively, Myo-Exo enhances the MMT and kidney fibrosis. Blockade of exosomes mediated myofibroblasts–macrophages communication may provide a novel therapeutic target for kidney fibrosis.

## 1. Introduction

Kidney fibrosis is a common outcome of chronic kidney disease (CKD), which is featured with exaggerated accumulation of extracellular matrix (ECM) and loss of renal function [1–3]. The cellular and molecular mechanism of kidney fibrosis is not fully elucidated. As available therapeutics is very limited for this devastating disorder, a deeper understanding of the cellular and molecular mechanism of kidney fibrosis might result in treatment strategies that delay progressive fibrosis [2,4].

Exosome is a nanoscale vesicle, which is secreted from cell to extracellular space by exocytosis [5,6]. Exosomes have recently emerged as active mediators of intercellular communication in multiple diseases [7,8]. It has been shown that renal tubule-derived exosomes promote fibroblasts activation and kidney fibrosis following either ischemic or obstructive injury [9,10]. Li et al. found that exosomes derived from mesenchymal stem cell attenuate renal injury [11]. Myofibroblasts are a key matrix-secreting cell type that plays a critical role in ECM accumulation in kidney fibrosis [12,13]. Recently, a study reveals that myofibroblast-derived exosomes (Myo-Exo) enhance cardiac endothelial cell dysfunction [14]. However, the role of Myo-Exo in renal fibrosis development is still unknown.

Myofibroblasts, featured with the expression of α-smooth muscle actin (α-SMA), is the major effector cell accounting for the production of ECM in the development of kidney fibrosis [12,15]. Myofibroblasts may originate from pericytes, fibroblasts, endothelial cells, and tubular epithelial cells (TECs) [13]. Macrophage is highly involved in renal fibrosis in both human and experimental diseases. Macrophage infiltration is a critical event in kidney fibrosis progression, which is usually related to the severity of fibrotic lesions [16]. Recently, accumulating data demonstrate that myofibroblasts originated from macrophages contribute profoundly to the pathogenesis of kidney fibrosis. Mounting evidence reveals that the transition of macrophages into myofibroblasts (MMT) plays a crucial role in renal fibrosis development [17–20]. Although multiple types of cells promote the progression of kidney fibrosis, MMT is an important event in the initiation and progression of this disease [20–22]. However, the role of Myo-Exo in the regulation of macrophages–myofibroblasts crosstalk and MMT process remains to be elucidated.

In the present study, we explored the effects of Myo-Exo on MMT and kidney fibrosis. Our results demonstrate that Myo-Exo mediate intercellular communication from myofibroblasts to macrophages and contribute significantly to MMT and kidney fibrosis progression.

## 2. Materials and methods

### 2.1. Mice information

The animal studies were conducted in accordance with the Guidelines of Laboratory Animal Care, and the experimental protocols involving animals were approved by the Animal Ethics Committee of Guangdong Zkenhealth Animal Center (approval no. SYXK 2021-0166). Male C57BL/6 mice (8–10 weeks old) were purchased from Guangdong Zhiyuan Biopharmaceutical Technology Co., Ltd. (Guangzhou, China). Mice were housed in pathogen-free and ventilated cages in a 12 h light/dark cycle, with room temperature at 25 ± 2 °C, and allowed free access to water and regular chow. For establishing a model of acute kidney injury (AKI) to CKD transition, mice were injected intraperitoneally with folic acid (FA) (250 mg/kg, Sigma, St. Louis, MO) [23]. In some experiments, exosomes were harvested from kidney fibroblasts stimulated with or without transforming growth factor-beta 1 (TGF-β1) and intravenously injected (500 µg per mouse). For exosome production inhibition, GW4869 (2.5 mg/kg per two days) is intraperitoneally injected [24]. For pharmacological inhibition of TANK-binding kinase 1 (TBK1), GSK8612 (1.5 mg/kg per two days) was intraperitoneally injected after FA treatment [23]. The mice were sacrificed at 14th day after FA injection. The kidneys were harvested and stored at −80 °C for further analysis.

### 2.2. Histology

Mouse kidneys were fixed in 10% formaldehyde, embedded in paraffin, and sectioned in 4 μm thickness for H&E or Sirius red staining. Evaluation of the fibrotic area was quantified with ImageJ software (NIH Image J system, Bethesda, MD).

### 2.3. Exosome isolation

Briefly, conditioned media (CM) of cell culture was centrifuged at 300 × *g* for 5 min, 2000 × *g* for 20 min, and 10,000 × *g* for 30 min at 4 °C to remove cells and cellular debris. Filtrate was subjected to ultracentrifugation at 110,000 × *g* for 1 h at 4 °C. This step was repeated by resuspending exosome pellet in sterile PBS. Finally, exosome pellets were resuspended in 50 µl PBS and stored at −80 °C till further use. After collecting the precipitate, ultracentrifugation was conducted again, and the supernatant without exosomes (exosome-depleted CM) was collected for follow-up experiments. The size and shape of exosomes were further measured by transmission electron microscopy (TEM) [25].

### 2.4. Cell culture and treatment

Primary kidney fibroblasts were isolated from mice as previously described [26]. Briefly, mouse kidney capsules were removed. The kidneys were then minced and digested with Liberase for 30 min at 37 °C. Cells were filtered through a 40 μm strainer, centrifuged, and cultured in DMEM containing 10% FBS and 1% penicillin and streptomycin. RAW264.7 cells were purchased from Procell Life Science & Technology Co., Ltd. (Wuhan, China).

For activating kidney fibroblasts, cells were treated with TGF-β1 (5 ng/ml) for 48 h. For labeling of exosomes, myofibroblasts were first labeled with PKH26 for 2 h and washed with PBS. PKH26-labeled exosomes from the CM of myofibroblasts were then incubated with RAW264.7 cells for 24 h or injected into the mice via the tail vein, and exosome distribution was determined with immunofluorescence staining.

In some experiments, fibroblasts were pretreated with GW4869 (20 µM). RAW264.7 macrophages were treated with CM of fibroblasts or exosomes (30 µg protein/ml) or GSK8612 (5 μM) for 24 h.

### 2.5. Immunofluorescence

Samples were then sectioned in 5 μm thickness. Sections and RAW264.7 cells cultured on coverslips for immunofluorescence were fixed with acetone, washed with PBS for three times, and blocked for 1 h. The sections were then incubated at 4 °C overnight with primary antibodies, including anti-fibronectin antibody (1:200, No. ab23750, Abcam, Cambridge, UK), anti-collagen 1 antibody (1:200, No. ab34710, Abcam, Cambridge, UK), and anti-α-smooth muscle actin antibody (1:200, No. A5228, Sigma, St. Louis, MO). The antibodies for double staining is anti-CD45 antibody (1:100, No. 610265, BD, Franklin Lakes, NJ), anti-F4/80 antibody (1:100, No. MCA497GA, Bio-Rad, Hercules, CA), anti-CD206 antibody (1:100, No. MCA2235, Bio-Rad, Hercules, CA), or anti-αSMA antibody (1:100, No. ab5694, Abcam, Waltham, MA). The immunofluorescence was detected using Alexa Fluor 488 antibody (Invitrogen, Carlsbad, CA) or Alexa Fluor 647 antibody (Invitrogen, Carlsbad, CA). Images were obtained on a fluorescence microscope equipped with a digital camera (Leica, Wetzlar, Germany) or confocal microscope (LSM 880, Zeiss, Jena, Germany). Measurement of the fluorescence staining area and count of positive cells was performed using ImageJ (NIH Image J system, Bethesda, MD).

### 2.6. Immunohistochemistry

Mouse kidneys were fixed in 10% formaldehyde and embedded in paraffin, and samples were then sectioned in 4 μm thickness. Using citrate buffer, sections were subjected to the antigen retrieval. For quenching of endogenous peroxidase activity, 3% hydrogen peroxide was used. Kidney sections were blocked, followed by incubation with phospho-TBK1 (1:100, No. 5483, Cell Signaling Technology, Boston, MA) antibody overnight at 4 °C. Sections were then incubated with secondary antibody. Positive staining was detected by incubating kidney sections with diaminobenzidine (DAB) solution. Nuclear staining was performed with hematoxylin. Images were photographed with microscope image system (Leica, Wetzlar, Germany).

### 2.7. Western blot analysis

Cells proteins or frozen kidney tissue proteins were extracted using lysis buffer containing protease and phosphatase inhibitors (Solarbio, Beijing Solarbio Science & Technology Co., Ltd., Beijing, China). The samples were run on SDS polyacrylamide gels of 8–15% depending on molecular weight of protein of interest. The separated proteins were then electrophoretically transferred onto nitrocellulose membranes. Thereafter, the membranes were probed with primary antibodies for CD63 (1:1000, No. ab315108, Abcam, Cambridge, UK), β-actin (1:5000, No. AC038, Abclonal, Woburn, MA), collagen 1 (1:1000, No. ab34710, Abcam, Cambridge, UK), α-SMA (1:1000, No. A5228, Sigma, St. Louis, MO), TBK1 (1:1000, No. 3504, Cell Signaling Technology, Boston, MA), and subsequently probed with the appropriate secondary antibody. Densitometry was performed using ImageJ software (NIH Image J system, Bethesda, MD).

### 2.8. Statistical analysis

All data were performed using SPSS version 24 (SPSS Inc., Chicago, IL). The data with normal distribution are presented as mean ± SEM. One-way ANOVA was used to compare groups, and Bonferroni’s test was performed for multiple comparisons. All tests were two tailed, and *p* values of <.05 were considered statistically significant.

## 3. Results

### 3.1. Myofibroblast-derived exosomes promote MMT *in vitro*

**Figure 1.**
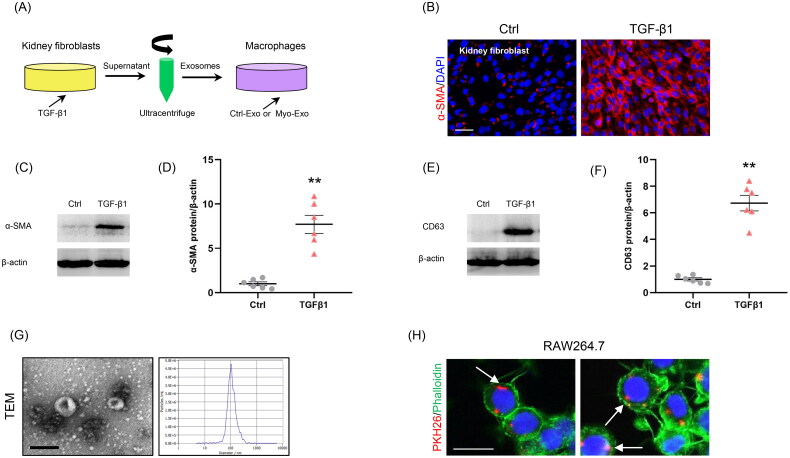
Myofibroblast-derived exosomes are associated with MMT. (a) Diagram depicting the experimental scheme. Kidney fibroblasts were treated with TGF-β1 (5 ng/ml) for 48 hours, PKH26-labeled exosomes from the conditioned media of myofibroblasts were incubated with cells for 24 h. (b) Immunostaining, scale bar, 50 μm. (c) Western blot shows the expressions of α-SMA. (d) Graphic presentation depicting the relative abundance for α-SMA normalized to β-actin in each group. (e) Western blot shows the expressions of CD63. (f) Graphic presentation depicting the relative abundance for CD63 normalized to β-actin in each group. (g) TEM displays the exosomes isolated from conditioned media of kidney fibroblasts stimulated by TGF-β1 (Myo-Exo). Scale bar, 200 nm. (h) Immunostaining depicting myofibroblast-derived exosomes (red) was taken up by macrophages. α-SMA: α-smooth muscle actin; MMT: transition of macrophages into myofibroblasts; PKH26: a specific dye for exosomes. **p <0.01 vs. Ctrl. *n* = 6 in each group.

To examine the role of Myo-Exo in MMT, exosomes from the CM of kidney fibroblasts stimulated without or with TGF-β1 (5 ng/ml) for 48 h were isolated (Figure 1(a)). Immunofluorescence staining and western blot showed that the α-SMA expressions were markedly increased in kidney fibroblasts treated with TGF-β1, confirming the transformation of fibroblasts to myofibroblasts (Figure 1(b–d)). TGF-β1 treatment led to a sharp increment of exosomal marker CD63 levels in kidney fibroblasts (Figure 1(e,f)). TEM exhibited majority of isolated extracellular vesicles being exosomes, as defined by their sizes (Figure 1(g)). We next labeled the Myo-Exo with PKH26, a specific dye for exosomes. As shown in Figure 1(h), when PKH26-labeled exosomes were incubated with RAW264.7 cells, they were up-taken by these cells, as outlined by staining for Phalloidin.

**Figure 2.**
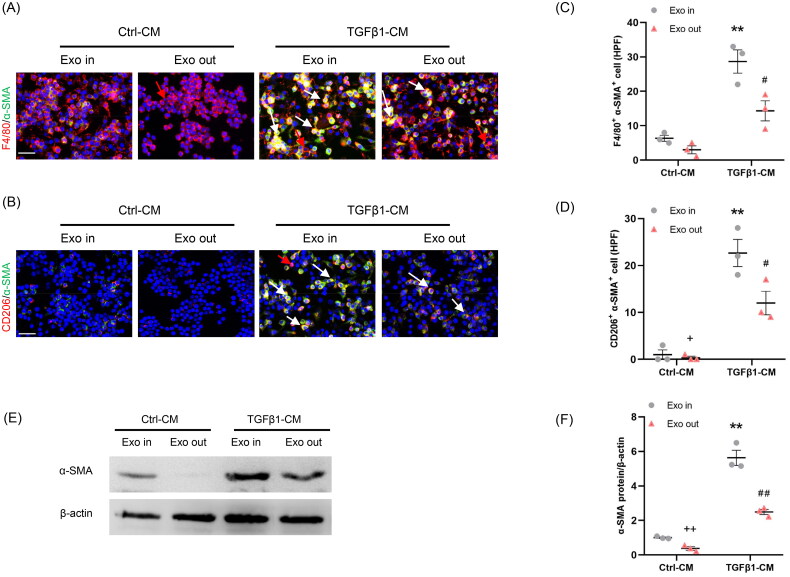
Myofibroblast-derived exosomes promote MMT *in vitro*. (a, b) Representative micrographs depicting the immunostaining for F4/80 or CD206 (red) and α-SMA (green) dual positive cells in cultured RAW264.7 cells exposed to exosomes isolated from kidney fibroblasts treated with or without TGF-β1. (c, d) Graphic presentation depicting the number of dual positive cells in each group. (e) Western blot depicting α-SMA abundance in RAW264.7 cells lysate, *n* = 6 in each group. (f) Graphic presentation depicting the relative abundance for α-SMA normalized to β-actin in each group. α-SMA: α-smooth muscle actin; MMT: transition of macrophages into myofibroblasts. White arrows represent double positive cells, red arrows represent single positive cells. Scale bar, 50 μm. ^+^*p* <0.05 or ^++^*p* <0.01 vs. TGFβ1-CM/Exo-out; **p <0.01 vs. Ctrl-CM/Exo-in; ^#^*p* <0.05 or ^##^*p* <0.01 vs. TGFβ1-CM/Exo-in. *n* = 3 in each group.

We next investigated whether Myo-Exo is essential for regulating crosstalk between myofibroblasts and macrophages. RAW264.7 cells were treated with complete CM (Exo In) or exosome-depleted CM (Exo Out). Our findings show that complete TGF-β1-CM treatment contributed significantly to the MMT in cultured macrophages. By contrast, exosome-depleted TGF-β1-CM treatment resulted in a sharp reduction of the number of F4/80^+^-α-SMA^+^ (F4/80, macrophage marker) or CD206^+^-α-SMA^+^ (CD206, M2 macrophage marker) cells in cultured macrophages (Figure 2). These data indicate that exosomes are required for regulating the communication of myofibroblasts–macrophages and MMT.

### 3.2. Inhibition of exosomes release impairs MMT *in vitro*

**Figure 3.**
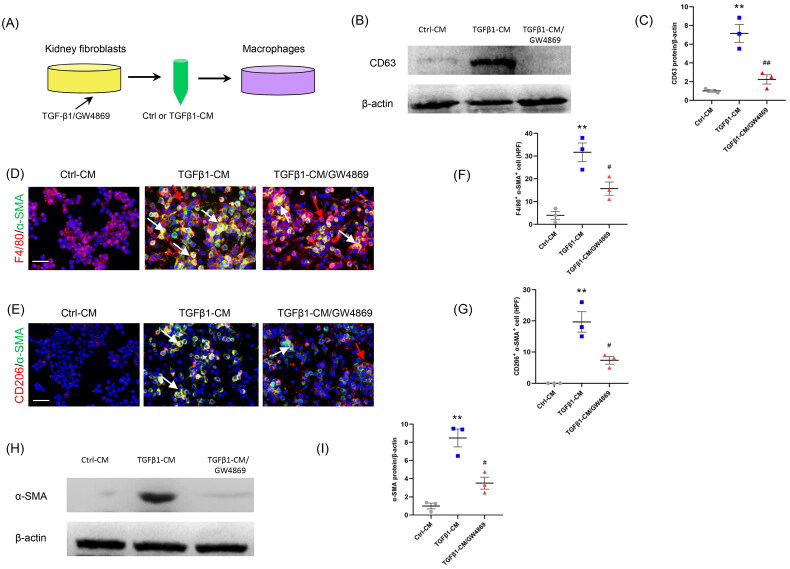
Inhibition of the exosome release from myofibroblasts impairs MMT *in vitro*. (a) Diagram depicting the experimental scheme. Kidney fibroblasts of mice were treated with TGF-β1 or GW4869. CM from kidney fibroblasts is harvested to stimulate RAW264.7 cells. (b) Western blot depicting CD63 abundance in CM of each group, *n* = 6 in each group. (c) Graphic presentation depicting the relative abundance for CD63 normalized to β-actin in each group. (d, e) Representative micrographs depicting the immunostaining for F4/80 or CD206 (red) and α-SMA (green) dual positive cells in cultured RAW.264.7 cells exposed to conditioned media from kidney fibroblasts treated with TGF-β1 or GW4869. (f, g) Graphic presentation depicting the number of dual positive cells in each group. (h) Western blot depicting and α-SMA abundance in RAW264.7 cells lysate. (i) Graphic presentation depicting the relative abundance for α-SMA normalized to β-actin in each group. CM: conditioned media; α-SMA: α-smooth muscle actin; MMT: transition of macrophages into myofibroblasts. White arrows represent double positive cells; red arrows represent single positive cells. Scale bar, 50 μm.**p <0.01 vs. Ctrl-CM; ^#^*p* <0.05 or ^##^*p* <0.01 vs. TGFβ1-CM. *n* = 3 in each group.

First, kidney fibroblasts of mice were treated with TGF-β1. Then, CM from kidney fibroblasts is harvested to stimulate RAW264.7 cells. We showed that there were more abundant F4/80^+^-α-SMA^+^ or CD206^+^-α-SMA^+^ cells in TGFβ1-CM group than those in Ctrl-CM group (Figure 3). These data suggest that CM from kidney fibroblasts treated with TGF-β1 triggered the MMT.

To investigate whether exosome inhibition suppresses the MMT, GW4869 (an exosome inhibitor) was used. Our results showed that administration of GW4869 significantly reduced the expression of CD63, a biomarker of exosomes (Figure 3(b,c)). Moreover, the number of F4/80^+^-α-SMA^+^ or CD206^+^-α-SMA^+^ cells was markedly decreased in GW4869 group (Figure 3(d–g)). Consistent with the results of immunofluorescence staining, western blot showed that the α-SMA protein levels were profoundly increased in TGFβ1-CM group compared to Ctrl-CM group (Figure 3(h,i)). Conversely, GW4869 treatment caused a sharp reduction of α-SMA protein levels. These data indicate that inhibition of exosomes blunts the MMT (Figure 3).

### 3.3. Myofibroblast-derived exosomes exacerbate kidney fibrosis in mice

We next evaluate whether Myo-Exo promote kidney fibrosis and ECM production. Exosomes isolated from TGF-β1-treated or Ctrl-treated kidney fibroblasts were injected into mice with FA injury (Figure 4(a,b)).

Figure 4.Myofibroblast-derived exosomes exacerbate kidney fibrosis in mice. (a) Diagram depicting the experimental scheme. Exosomes isolated from TGF-β1-treated or Ctrl-treated kidney fibroblasts were injected into mice with FA injury. (b) Representative micrographs depicting the presence of PKH67-labeled exosomes in macrophages of mouse kidney. Scale bar, 200 nm. (c) H&E staining and (d) Sirius red staining depicting renal injury or collagen deposition in kidneys of mice in each group. (e, f) Graphic presentation depicting renal injury or collagen deposition in kidneys of mice in each group. (g, h) Representative micrographs. (i, j) Graphic presentation depicting the immunostaining for FN (green) and collagen 1 (green) in kidneys of mice in each group. (k) Western blot depicting collagen 1 abundance in in kidneys lysate. (l) Graphic presentation depicting the relative abundance for collagen 1 normalized to β-actin in each group. Scale bar, 50 μm. ^+^*p* <0.05 or ^++^*p* <0.01 vs. FA/Myo-Exo; **p <0.01 vs. Sham or Sham/Ctrl-Exo; ^##^*p* <0.01 vs. FA or Sham/Myo-Exo. *n* = 6 in each group.

**Figure:**
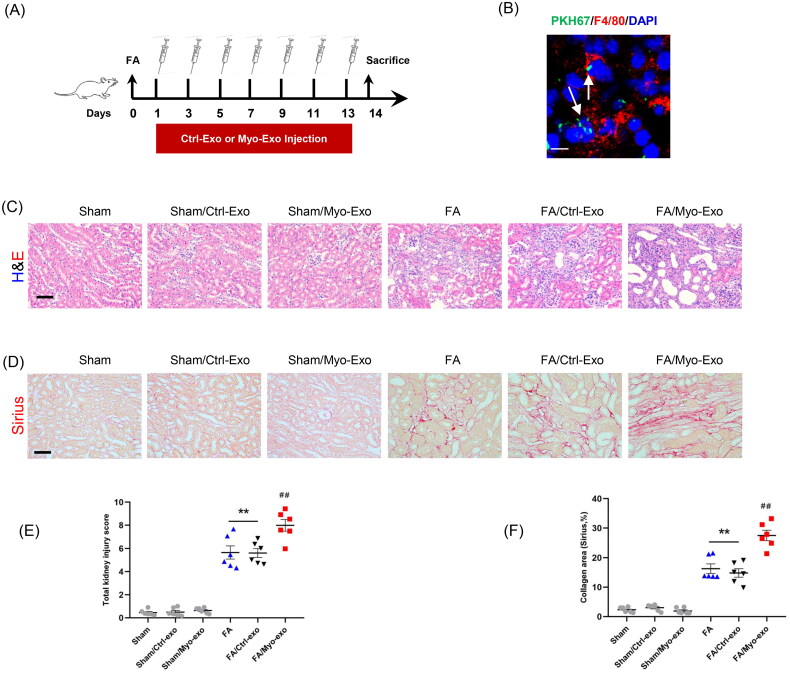

**Figure:**
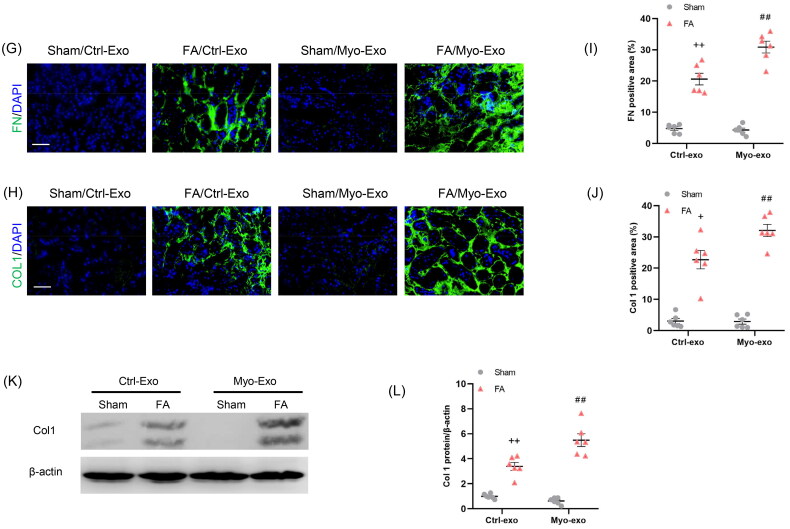

H&E and Sirius staining displayed that Myo-Exo treatment exacerbated renal injury and collagen deposition in injured kidneys of mice compared to Ctrl-Exo mice. Of note, H&E and Sirius staining showed that Myo-Exo or Ctrl-Exo by itself had no effects on kidneys of sham mice (Figure 4(c–f)). Consistent with above-mentioned results, immunofluorescence staining revealed that Myo-Exo promoted the expressions of fibronectin and collagen I proteins in injured kidneys of mice compared to Ctrl-Exo mice (Figure 4(g–l)). These data suggest that myofibroblast-derived exosomes contribute profoundly to kidney fibrosis in FA nephropathy.

### 3.4. Myofibroblast-derived exosomes aggravate MMT and myofibroblast accumulation in mice

**Figure 5.**
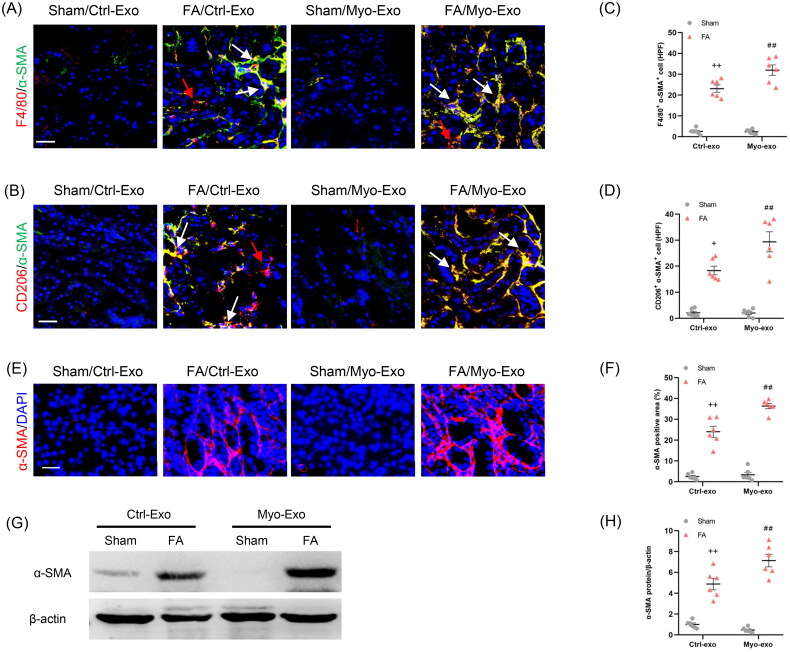
Myofibroblast-derived exosomes promote MMT and myofibroblast accumulation in kidneys of mice. (a, b) Representative micrographs depicting the immunostaining for F4/80 or CD206 (red) and α-SMA (green) dual positive cells in kidneys of mice in each group. (c, d) Graphic presentation depicting the number of dual positive cells in each group. (e) Representative micrographs and (f) graphic presentation depicting the immunostaining for α-SMA (red) in kidneys of mice in each group. (g) Western blot depicting α-SMA abundance in kidneys lysate. (h) Graphic presentation depicting the relative abundance for α-SMA normalized to β-actin in each group. CM: conditioned media; α-SMA: α-smooth muscle actin. White arrows represent double positive cells, red arrows represent single positive cells. ^+^*p* <0.05 or ^++^*p* < 0.01 vs. FA/Myo-Exo; ^##^*p* < 0.01 vs. Sham/Myo-Exo. Scale bar, 50 μm, *n* = 6 in each group.

To evaluate whether Myo-Exo promote MMT, kidney sections were stained for F4/80 and α-SMA. We showed that Myo-Exo treatment increased MMT cells in injured kidneys, as indicated by more F4/80 and α-SMA dual positive cells compared to Ctrl-Exo mice (Figure 5(a,c)). Furthermore, Myo-Exo treatment significantly augmented CD206 and α-SMA dual positive cells in kidneys of mice with FA injury than that in Ctrl-Exo mice (Figure 5(b,d)). Given that M2 macrophages have a critical role in initiating fibrotic development [27,28], we examine the effects of Myo-Exo on M2-MMT. The data indicate that Myo-Exo aggravates MMT in FA induced kidney injury.

In agreement with above-mentioned results, immunofluorescence staining and western blot results displayed that Myo-Exo treatment led to a sharp increment of α-SMA protein levels in injured kidneys compared with mice treated with Ctrl-Exo (Figure 5(e–h)). These results indicate that exosomes derived from myofibroblasts aggravate myofibroblast accumulation in FA nephropathy.

### 3.5. Inhibition of exosomes protects against kidney fibrosis in mice

**Figure 6.**
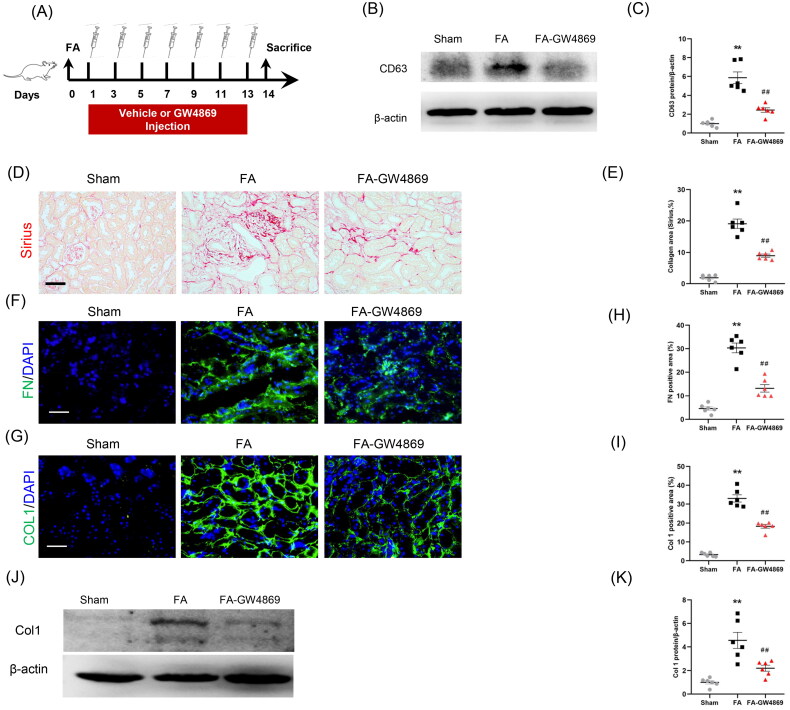
Inhibition of exosomes ameliorates kidney fibrosis in mice. (a) Diagram depicting the experimental scheme. Mice were subjected to FA injection for 14 days to induce kidney fibrosis with vehicle or GW4869 injection. (b) Western blot depicting CD63 abundance in kidneys of each group. (c) Graphic presentation depicting the relative abundance for CD63 normalized to β-actin in each group. (d) Sirius red staining and (e) graphic presentation depicting collagen deposition in kidneys of mice in each group. (f, g) Representative micrographs. (h, i) Graphic presentation depicting the immunostaining for FN (green) and collagen 1 (green) in kidneys of mice in each group. (j) Western blot depicting collagen 1 abundance in kidneys lysate. (k) Graphic presentation depicting the relative abundance for collagen 1 normalized to β-actin in each group. GW4869: inhibition of exosome production; CD63: exosomal marker. **p < 0.01 vs. Sham; ^##^*p* < 0.01 vs. FA. Scale bar, 50 μm, *n* = 6 in each group.

We next investigated the effects of exosome inhibition on kidney fibrosis in a mouse model of FA nephropathy. Mice were subjected to FA injection for 14 days to induce kidney fibrosis (Figure 6(a)). Sirius red staining was performed to examine collagen deposition in the kidneys (Figure 6(d,e)). GW4869 was used to inhibit exosome secretion. As shown in Figure 6, CD63 levels in kidneys with FA injury were significantly increased, whereas GW4869 treatment caused a notable reduction of CD63 levels. GW4869 treatment reduced collagen deposition in the kidneys with FA injury (Figure 6(b,c)). Immunofluorescence staining displayed that protein levels of fibronectin and collagen I in FA-injured kidneys were markedly decreased in mice treated with GW4869 compared with sham mice (Figure 6(f–k)). These observations suggest that exosomes has an important role in ECM protein accumulation and kidney fibrosis in FA nephropathy.

### 3.6. Inhibition of exosomes blunts MMT and myofibroblast accumulation in mice

**Figure 7.**
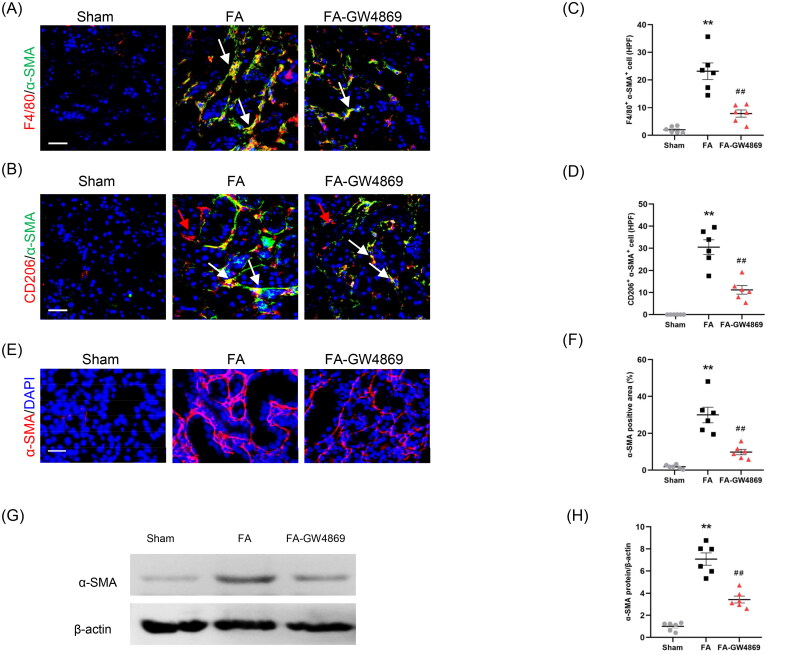
Inhibition of exosomes blunts MMT and myofibroblast accumulation in kidneys of mice. (a, b) Representative micrographs depicting the immunostaining for F4/80 or CD206 (red) and α-SMA (green) dual positive cells in kidneys of mice in each group. (c, d) Graphic presentation depicting the number of dual positive cells in each group. (e) Representative micrographs and (f) graphic presentation depicting the immunostaining for α-SMA (red) in kidneys of mice in each group. (g) Western blot depicting α-SMA abundance in kidneys cells lysate. (h) Graphic presentation depicting the relative abundance for α-SMA normalized to β-actin in each group. α-SMA: α-smooth muscle actin. White arrows represent double positive cells, red arrows represent single positive cells. **p < 0.01 vs. Sham; ^##^*p* < 0.01 vs. FA. Scale bar, 50 μm, *n* = 6 in each group.

To examine whether exosome inhibition blunts MMT, kidney sections were stained for F4/80 and α-SMA. Our findings showed that inhibition of exosomes decreased MMT cells in injured kidneys, as indicated by fewer F4/80 and α-SMA dual positive cells (Figure 7(a,c)). In terms of M2 macrophage is the predominant type cell in the process of MMT, we evaluated the effects of exosome inhibition on the transformation of M2 macrophages to myofibroblasts. We showed that inhibition of exosomes considerably reduced CD206 and α-SMA dual positive cells in kidneys of mice with FA injury (Figure 7(b,d)). These results suggest that exosome has an important role in mediating the process of MMT.

We next examined the effect of exosome inhibition on myofibroblast accumulation. Immunofluorescence staining and western blot results showed that GW4869 treatment caused a significant reduction of α-SMA protein levels in the injured kidneys compared with mice with FA injury (Figure 7(e–h)). These results indicate that inhibition of exosomes decreases myofibroblast accumulation in the injured kidney of mice subjected to FA treatment.

### 3.7. TBK1 inhibitor abolished the profibrotic effects of myofibroblast-derived exosomes

**Figure 8.**
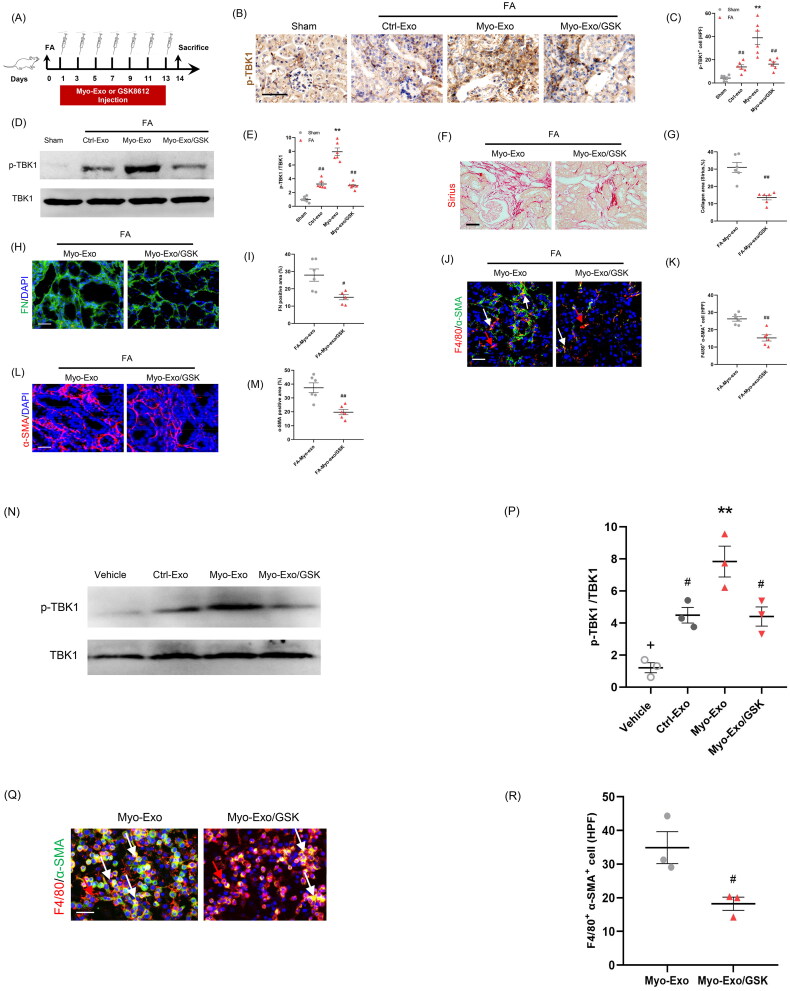
TBK1 inhibitor abolished the profibrotic effects of myofibroblast-derived exosomes. (a) Diagram depicting the experimental scheme. (b) Representative micrographs depicting the immunostaining for phosph-TBK1 positive cells in kidneys of mice in each group. (c) Graphic presentation depicting the number of phosph-TBK1 positive cells in each group. (d) Western blot depicting TBK1 abundance in kidneys cells lysate. (e) Graphic presentation depicting the relative abundance for p-TBK1 normalized to TBK1 in each group. (f) Sirius red staining. (g) Representative micrographs. (h) Graphic presentation depicting the immunostaining for FN (green) in kidneys of mice in each group. (i) Representative micrographs. (j) Representative micrographs depicting the immunostaining for F4/80 (red) and α-SMA (green) dual positive cells in kidneys of mice in each group. (k) Graphic presentation depicting the number of dual positive cells in each group. (l) Graphic presentation depicting the immunostaining for α-SMA (red) in kidneys of mice in each group. (m) Representative micrographs. (n) Western blot depicting TBK1 abundance in RAW.264.7 cells. (p) Representative micrographs (q) Representative micrographs depicting the immunostaining for F4/80 (red) and α-SMA (green) dual positive cells in cultured RAW.264.7 cells exposed to Myo-Exo or GSK in each group. (r) Representative micrographs. α-SMA: α-smooth muscle actin. White arrows represent double positive cells, red arrows represent single positive cells. Scale bar, 50 μm. ^+^*p* < .05 vs. FA/Myo-Exo/GSK or Myo-Exo/GSK; **p < 0.01 vs. Sham or FA/Myo-Exo or vehicle; ^#^*p* < 0.05 or ^##^*p* < 0.01 vs. FA/Myo-Exo or Myo-Exo. Scale bar, 50 μm, *n* = 6 in each group.

TANK-binding kinase 1 has a crucial role in the regulation MMT and renal fibrosis [23]. We next explored whether the profibrotic effects of Myo-Exo was involved in TBK1. We showed that Myo-Exo treatment substantially enhanced TBK1 activation levels in FA-injured kidneys compared to FA treatment alone. Conversely, GSK (a TBK1 inhibitor) treatment led to a significant reduction of TBK1 phosphorylation levels (Figure 8(b–e)). Sirius and immunofluorescence staining exhibited that Myo-Exo treatment augmented collagen deposition and ECM production in FA-injured kidneys, which was largely abolished by GSK treatment. Consistent with these results, Myo-Exo treatment promoted MMT and myofibroblast accumulation in FA-injured kidneys, which was largely negated by GSK treatment (Figure 8(e–m)). In addition, we obtained the same results *in vitro*, Myo-Exo treatment accelerated MMT and up-regulated the expression level of p-TBK1 in RAW264.7 cells, which was significantly reversed by GSK treatment (Figure 8(n–r)). These data suggest that the profibrotic effects of myofibroblast-derived exosomes might be associated with TBK1 signaling.

## 4. Discussion

Exosomes have been documented to play a critical role in kidney fibrosis progression by intercellular crosstalk [9,29–32]. Nevertheless, the role of myofibroblast-derived exosomes in the pathogenesis of kidney fibrosis remains unclear. In this study, we demonstrate that exosome derived from myofibroblast has an important role in the regulation of renal fibrosis development. The main findings are as follow: (1) myofibroblast-derived exosomes contribute to MMT and kidney fibrosis; (2) pharmacological inhibition of exosomes attenuates MMT and kidney fibrosis; (3) the profibrotic effects in kidneys of myofibroblast-derived exosomes might be involved in TBK1 signaling. These findings present novel insights on exosomes mediating the communication between myofibroblasts and macrophages in kidney fibrosis development.

Exosomes are membrane-bound tiny biological particles ranging from about 30 to 150 nm in diameter. Exosomes are released virtually by most cell types and extensively participate in numerous pathological and physiological processes in various organs [33,34]. In early years, it is considered that exosomes shift excess or unnecessary constituents from cells to maintain cellular homeostasis, functioning like garbage bags [35]. Recent evidence shows that exosomes play a crucial role in mediating cell-to-cell crosstalk and influence the phenotype of cells which they fuse or interact with [36–39]. Accumulating data indicate that exosomes exert vital effects on fibrotic disorder, including renal fibrosis [9,40–42]. Myofibroblasts are critical effector cells responsible for dysregulated ECM deposition in fibrosis process [12]. However, the role of myofibroblast-derived exosomes in kidney fibrosis is not established. In this study, we first investigate the impact of myofibroblast-derived exosomes on ECM production and renal fibrosis induced by FA. Moreover, pharmacological inhibition of exosomes significantly reduces collagen accumulation and fibroblast activation, thus protects against the development of kidney fibrosis in FA nephropathy.

After kidney injury, macrophages infiltrate the kidney and differentiate into M2 macrophages in response to persistent inflammation, and then transition to myofibroblasts that contributes to kidney fibrosis progression [43]. This process is termed as the MMT. MMT cells co-express macrophage markers, such as F4/80, and myofibroblast markers, such as α-SMA [44]. Our team previously demonstrates that MMT process plays an important role in FA, obstructive injury, and hypertensive nephropathy [17,43]. To further investigate whether the profibrotic effects of exosomes are involved in MMT, co-staining for F4/80 and α-SMA is used to identify MMT cells in kidney tissues or cultured cells. Our findings reveal that myofibroblast-derived exosome treatment leads to a significant increase of F4/80^+^-αSMA^+^ cells and contributes to the M2-MMT *in vivo* or *in vitro*. Conversely, pharmacological inhibition of exosomes markedly reduces the number of F4/80^+^-αSMA^+^ cells and precludes the M2-MMT in injured kidneys of mice or cultured macrophages. These data indicate that MMT might mediate the profibrotic effects of myofibroblast-derived exosomes on kidneys with FA injury.

TBK1, an IKK-related kinase along with IKKε, has critical roles in the regulation of immune responses, autophagy, and energy metabolism [45]. Numerous studies have shown that TBK1 signaling is closed related to fibrotic diseases [46–49]. Our team has demonstrated that pharmacological inhibition of TBK1 attenuates MMT process and kidney fibrosis [23]. Therefore, we investigate whether the profibrotic effect of Myo-Exo is associated with TBK1. The positive findings we obtained, which are consistent with previous research, suggest that TBK1 signaling might mediate the profibrotic effect of Myo-Exo.

Accumulating data reveal that exosome-based cells–cells communication depends on the tiny biological cargos they delivered, such as microRNA and circular RNA [33,34,50]. In this study, we did not investigate which biological molecules myofibroblast-derived exosomes contain. This is a limitation of our study. Further investigation is required to explore exosome miRNA-mediated network communication between myofibroblasts and macrophages. Furthermore, we focused on the relationship between exosomes and macrophages in the current research. We did not perform co-staining PKH67 and other cells marker such as vimentin. Therefore, the possibility of exosomes acting on fibroblasts or TECs cannot be excluded. This is another limitation of our study.

**Figure 9.**
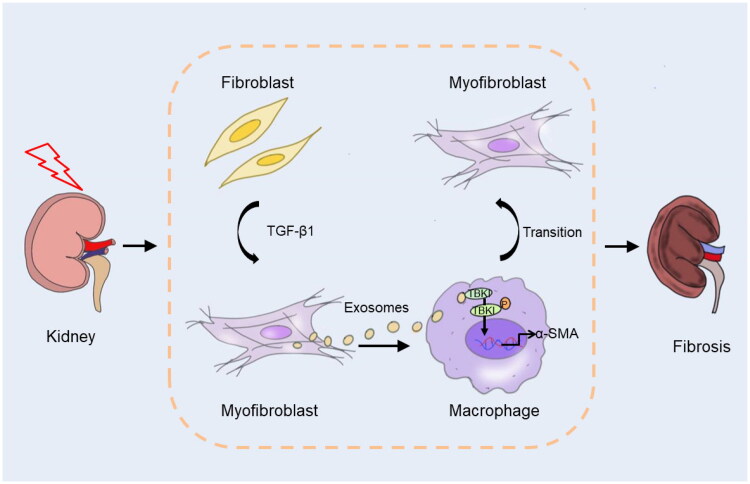
Graphical abstract. In response to stress, kidney fibroblasts differentiate into myofibroblasts. Exosomes derived from myofibroblasts activate TBK1 in macrophage, resulting in macrophage to myofibroblast transition and kidney fibrosis.

Taken together, we elucidate a new molecular mechanism that myofibroblast-derived exosome is an important participant in MMT and kidney fibrosis progression via mediating crosstalk between myofibroblasts and macrophages. In response to stress, kidney fibroblasts differentiate into myofibroblasts. Exosomes derived from myofibroblasts activate TBK1 in macrophage, resulting in MMT and kidney fibrosis (Figure 9). Blockade of exosomes mediated myofibroblasts–macrophages communication may provide a novel therapeutic target for kidney fibrosis.

## Data Availability

The data used to support the findings of this study are available from the corresponding authors upon request.
